# A Simple Method to Reduce both Lactic Acid and Ammonium Production in Industrial Animal Cell Culture

**DOI:** 10.3390/ijms19020385

**Published:** 2018-01-28

**Authors:** Nathaniel W. Freund, Matthew S. Croughan

**Affiliations:** 1 Current address: Kite, a Gilead Company, Santa Monica, CA 90404, USA; nfreund@kitepharma.com; 2 Amgen Bioprocessing Center, Keck Graduate Institute, Claremont, CA 91711, USA

**Keywords:** lactic acid, ammonium, animal cell culture, industrial, fed-batch, recombinant proteins

## Abstract

Fed-batch animal cell culture is the most common method for commercial production of recombinant proteins. However, higher cell densities in these platforms are still limited due to factors such as excessive ammonium production, lactic acid production, nutrient limitation, and/or hyperosmotic stress related to nutrient feeds and base additions to control pH. To partly overcome these factors, we investigated a simple method to reduce both ammonium and lactic acid production—termed Lactate Supplementation and Adaptation (LSA) technology—through the use of CHO cells adapted to a lactate-supplemented medium. Using this simple method, we achieved a reduction of nearly 100% in lactic acid production with a simultaneous 50% reduction in ammonium production in batch shaker flasks cultures. In subsequent fed-batch bioreactor cultures, lactic acid production and base addition were both reduced eight-fold. Viable cell densities of 35 million cells per mL and integral viable cell days of 273 million cell-days per mL were achieved, both among the highest currently reported for a fed-batch animal cell culture. Investigating the benefits of LSA technology in animal cell culture is worthy of further consideration and may lead to process conditions more favorable for advanced industrial applications.

## 1. Introduction

### 1.1. Summary of Methods to Reduce Lactic and Ammonium Production

In the field of animal cell culture, many researchers have observed lactic acid and/or ammonium accumulate to levels that inhibit cell growth and/or productivity [1,2,3,4,5,6,7,8,9,10,11,12]. For over 60 years, a number of methods have been developed to minimize lactic acid and/or ammonium production [1,2,3,4,10,11,12,13,14,15,16,17,18,19,20,21,22,23,24,25,26,27,28,29,30,31,32,33,34,35,36,37,38]. A summary of the common methods is shown in Table 1. Over roughly the last 35 years, the challenges regarding lactic acid and ammonium accumulation have substantially increased, as the biopharmaceutical industry and its partners in academia have moved from low density batch culture to high density fed-batch culture [3,4,5,6,10,11,12,24,27,28,29,30]. An ideal method for use in the manufacture of human therapeutics through industrial animal cell culture would:

(1) Be universally effective—across all industrially-relevant cell lines and processes—at reducing both lactic acid and ammonium production to sufficiently low levels so as to have no negative impact on cell growth and product quality,
(2) Lead to no increase and possibly even a decrease in process complexity, as discussed further below, and
(3) Be commonly implemented in large-scale industrial operations run according to current Good Manufacturing Practices (cGMPs), with a solid track record of success over many years.

As shown in Table 1 and discussed further below, none of the current methods meet all three of these criteria.

Many of the earliest methods shown in Table 1, such as replacement of glucose with alternative sugars, were developed to reduce lactic acid accumulation. At the time, it was not widely recognized that ammonium accumulation could be an issue, and thus data regarding ammonium levels was not reported (NR). In fact, many of the earliest methods, along with many of the current methods, reduce lactic acid production but increase ammonium production or, at best, certainly have not been proven to universally reduce ammonia production. When tested for use in fed-batch cultures, alternative sugars such as galactose sometimes lead to similar growth as with glucose [3] but often do not [27].

Relatively low levels of ammonium, typically between 2 to 10 mM, may have negative effects on cell growth and productivity, as well as the glycosylation and possibly even the biological activity, stability, and/or immunogenicity of certain recombinant glycoproteins [4,39,40,41,42,43,44,45,46]. Changes in post-translational processing are believed to be related to intracellular pH increases, which are brought on by ammonia transport into the Golgi [41] and/or energy depletion [47]. For these reasons, ammonium is one of the most important inhibitory metabolites in animal cell culture [48]. Negative effects from ammonium are often attained at concentrations approximately ten-fold less (e.g., 2–4 mM) than for lactate (e.g., 20–40 mM) [42,43,49]. Thus, decreasing lactic acid at the expense of even a small increase in ammonia is certainly not desirable.

Control of glucose at low levels in the culture medium—so as to limit the supply of glucose to the cells—has been employed for over three decades to reduce lactic acid production (Table 1). This can be achieved through various means, such as direct measurement and automatic or manual feedback control of glucose levels. When glucose but not glutamine is controlled at low levels, a desirable decrease in glucose uptake and lactic acid production typically occurs, but this often goes hand-in-hand with an undesirable increase in glutamine uptake and ammonium production [22,23,24,27,50]. Thus, to minimize both lactic acid and ammonium production, glucose as well as glutamine may need to be simultaneously controlled [4,24,25,45] or replaced [20].

### 1.2. Process Complexity and Lack of Industrial Implementation 

Since the time of the pioneering work of Fleischaker [3]—wherein it was found that glucose levels of as low as 0.1 mM were needed to largely eliminate lactic acid production—there have been several refinements in the systems used for automatic feedback control of glucose and/or glutamine. This has resulted in a long series of interesting publications from academic or industrial process development groups. That said, many feedback control approaches require the following four components:

(1) Frequent sample withdrawal, often using an automatic sampling system,
(2) Sample testing for glucose and/or glutamine levels using an external sensor system,
(3) Transmission of the test result into a computer control system for processing, and
(4) Frequent culture additions from a glucose and/or glutamine reservoir, using a pump and transfer line.

When one adds the four components above to a culture system, there is a very substantial increase in process complexity. 

Although complex systems with the four components above have been successfully used many times in laboratory-scale bioreactors, and have repeatedly shown substantial benefits at the lab scale in both academia and industry, they have never, to our knowledge, been implemented as part of a licensed, large-scale animal cell culture process. Even for *E. coli* production cultures, with durations of only 1–2 days, there are substantial challenges around continuous feeding of nutrients in large-scale, cGMP operations [51,52]. For animal cell production cultures, with durations that are typically at least 10–15 days, these challenges increase, as the system must perform continuously without problems for a much longer period. The chance of run failure is considered too high, not only due to the complexity of the system, but also due to the resulting risks around contamination and robust feedback control at “near failure” nutrient levels. Glucose depletion can lead to apoptosis and premature cell death [53] or affect product quality by reducing glycosylation [39,54]. Accordingly, glucose levels for most industrial fed-batch processes are held above 1 g/L or higher [31,38], well above the much lower range required to reduce lactic acid production. 

A recent approach, coined HI-end pH-controlled Delivery of Glucose (HIPDOG) by Gagnon et al. [27], has been shown to dramatically reduce lactic acid production and also substantially increase titers without the use of an external sensor system and frequent sample withdrawal. This strategy relies on the pH control loop to deliver glucose when the pH rises. The method requires the use of a pH sensor, feed transfer line, pump, and glucose feed reservoir for every culture, adding to the complexity of each culture system. It is thus quite difficult to implement for a large number of very small-scale cultures, such as those used for cell line screening. However, it does not require frequent sampling of culture fluid for glucose and/or glutamine analysis and thus does not add those associated contamination and sensor failure risks. For large-scale cultures, the increase in performance provided by HIPDOG is apparently worth the increase in complexity. It has been implemented in industrial cGMP cell cultures, has been used to substantially improve legacy processes, and has provided some of the best published fed-batch culture performance to date. There are no published reports of implementation by firms other than Pfizer. Like many other low-glucose control systems, however, the approach results in an increase in peak ammonium levels [27]. The success of the HIPDOG approach may thus be enhanced if used in combination with Glutamine Synthetase transfected Chinese Hamster Ovary (GS-CHO) lines. Glutamine synthetase (GS) transfection works with both CHO and NSO lines [55] and may well work universally. It not only provides cell lines with high specific productivities, but is also a metabolic engineering method to reduce ammonia production [56,57]. When used in combination with HIPDOG, GS technology may often keep ammonium within acceptable ranges. 

There are also other approaches to dynamic nutrient feeding, such as ones that rely on the frequent measurement of oxygen uptake rate and numerous other culture parameters [3,28]. These measurements are used in combination with various stoichiometric and/or other mathematical models to determine optimum feed quantities and/or formulations. Although these methods do not require frequent sampling for measurement and feedback control of glucose and/or glutamine, they still add a substantial degree of process complexity, and are thus rarely if ever fully implemented in cGMP operations. Certain aspects, such as stoichiometric design of medium and feeds, are commonly employed in modern processes. 

### 1.3. Metabolic Engineering 

Many researchers have attempted to develop metabolic engineering methods to reduce lactic acid and/or ammonium production. To limit the scope of this introduction, these methods are not cited in Table 1. None meet all three criteria specified in the first paragraph of this subsection. The reader is referred to Young [58], Kim et al. [59], and Dietmair et al. [60], who all present excellent reviews and analyses of these methods. In general, improvement of metabolic phenotypes through genetic engineering has proven more difficult than originally envisioned back in the 1980’s. Beyond the GS approach, none of the other metabolic engineering methods to reduce lactic acid and/or ammonia production have found widespread adoption in industry to date [58,60,61]. 

### 1.4. Common Simple Methods 

Lastly, Table 1 presents four simple and common methods to reduce lactic acid and/or ammonium production: (1) lowering culture temperature, (2) lowering culture pH, (3) supplementation of the medium with copper, and (4) selection of clones with lactate-consumption (LC) phenotype. Although all of these methods are commonly employed, none have been proven to universally reduce both lactic acid and ammonia production. Temperature and pH shifts are generally optimized on case-by-case basis and can negatively or positively impact cell growth and/or product quality [10,11,27,33,34,35,36,37,38,62]. Although a drop in pH will very often reduce lactic acid production [35,36,37,63,64], it can also lead to an increase in glutamine consumption and ammonium production [37]. Copper supplementation is a simple approach but does not reduce lactic acid production for all relevant cell lines [12,30] and can also positively or negatively impact product quality [12,29,31,65,66]. Selection of clones with a lactate-consumption (LC) phenotype is inherently not universal, as the clone with the best specific productivity, product quality, or otherwise greatest potential might be discarded due to lack of the LC phenotype. For certain clones, the LC phenotype may be occasionally lost due to unknown factors, even in validated cGMP processes making commercial products [11]. Such changes in phenotype are reasonably common but rarely reported in the literature [67]. Problems persist [11,68] even though a combination of approaches to maximize cell growth and productivity while minimizing lactic acid and/or ammonium production, such as stoichiometric design of medium and feeds, have been employed for over 35 years [3,4,5,6,26,28,38,42,43,44,45,46,68,69,70,71,72,73,74]. 

### 1.5. Lactate Supplementation and Adaptation (LSA) Technology

In response to the limitations of the methods discussed above, a doctoral research project was undertaken to invent and develop a new method to reduce both lactic acid and ammonium production in industrial animal cell culture. Ideally, it would be a simple, robust method that could be readily and quickly employed across multiple scales, from small multi-well plate cultures used for cell line screening to large stirred tank cultures used for commercial manufacturing. Ideally, it would not increase or possibly even decrease culture complexity, with no need for additional equipment, such as that needed for new feed-back control loops. Ideally, it would work universally for all relevant cell lines and processes. This paper covers the initial feasibility and proof-of-concept studies for this new method. 

Our new method was inspired partly by the observation that many fed-batch processes exhibit periods of lactate consumption or no net lactate production [11,12,27,30,32,38,50,70,75,76,77,78]. These periods generally occur later in culture, after the exponential growth phase, often after lactate has accumulated to levels of at least 2–4 g/L or higher. The goal was to extend this behavior throughout the culture, including the exponential growth phase. 

Conceptually, the overall production of lactic acid from glucose can be written as [79]:

Glucose + 2 HPO_4_^−2^ + 2 ADP → 2 Lactate^−^ + 2 H^+^ + 2 ATP (1)

Lactic acid has a p*K*a value of 3.87 and will dissociate under physiological conditions to lactate and a hydrogen molecule. Although the entire reaction path to lactate involves multiple steps through glycolysis, a branch point exists at pyruvate, which can either flux toward the tricarboxylic acid (TCA) cycle for the continued production of ATP or be reduced to lactate and contribute to culture acidity [80], as shown in Equation (2):

Pyruvate^−^ + NADH + H^+^ ↔ Lactate^−^ + NAD^+^ (2)

Although the equilibrium balance highly favors lactic acid production (∆G′° = −25.1 kJ/mol) this flux can hypothetically be stopped or reversed by high levels of lactate as explained by the actual free energy change, ∆G, of the reaction [80], as shown in Equation (3):

(3) $$\Delta G=\Delta G^{\prime }{}^{\circ}+RT\ln\left(\frac{\left[{\mathrm{LAC}}^{-}\right]\cdot \left[{\mathrm{NAD}}^{+}\right]}{\left[{\mathrm{PYR}}^{-}\right]\cdot \left[\mathrm{NADH}\right]}\right)$$

where

(4) $$\Delta G^{\prime }{}^{\circ}=RT\ln K_{eq}^{\prime }=-25.1\;\mathrm{kJ}/\mathrm{mol}$$

Note that H^+^ is not included in the ratio according to convention [81].

Our proposed mechanism of action, as well as the rationale behind our approach, centers on the law of mass action. Lactate concentrations that increase the stoichiometric ratio in Equation (3) so as to match the equilibrium constant in Equation (4), *K*′*_eq_*, should generate a zero ∆G and eliminate net lactate production. This approach rests on the assumptions that (1) increased lactate levels in the medium lead to increased lactate levels inside the cell (i.e., higher intracellular lactate), and (2) in response to increases in intracellular lactate, the stoichiometric ratio in Equation (4) will increase, even though the levels of the four individual chemical species may change. If these assumption are true, the reaction path from pyruvate to lactate can potentially be controlled over one reaction, via simple lactate supplementation of the culture medium, even though other phenomena may be coming into play, such as inhibition of phosphofructokinase by lactate. 

Ideally, an increase in intracellular lactate will not only increase the stoichiometric ratio in Equation (3), but also increase levels of sodium pyruvate as well as the flux of pyruvate into the TCA cycle [79]. If so, it could lead to increased levels of TCA cycle intermediates, such as α-ketoglutarate, and possibly reduce the production of ammonium, again through the laws of mass action, by slowing or eliminating the conversion of glutamate to α-ketoglutarate via glutamate dehydrogenase. Alternatively or in addition, increased levels of intracellular pyruvate may slow or eliminate the conversion of alanine to pyruvate via alanine dehydrogenase, and as proposed by Li et al. [38], thereby reduce ammonium production. This possible mechanism is again based upon the law of mass action.

In support of this approach, others have separately found that lactate supplementation can reduce lactate production [7,8] and, even when targeted at relatively low levels, can reduce ammonium production [38].

The proposed mechanisms of action presented above, centered on the laws of mass action, are provided only to show the rationale behind our proposed new method. They will not be directly investigated per se in this paper. Many others have investigated the mechanism(s) of action behind metabolic shifts from lactate production to lactate consumption [30,32,38,55,82]. If the results of this feasibility study are promising, similar mechanisms of action studies would be appropriate for our new method. 

To apply this new method, cultures are first inoculated into fresh medium supplemented with various levels of sodium lactate. The changes in lactate levels are monitored. A sufficient level of lactate supplementation is identified that nearly or totally eliminates the net production of lactate, i.e., holds lactate levels constant. Cells are then grown in that level of lactate for multiple passages until their growth rate matches that of the original cells grown in medium not supplemented with lactate. 

In previous studies, lactate supplementation of medium has been performed to study the impact of lactate on cell growth and/or productivity [7,8,27,83], to derive lactate-tolerant cell lines [84], or to eliminate lactate depletion during the lactate consumption phase [38]. Lactate supplementation levels in these studies were selected to reflect maximum or normal levels that arise naturally in a culture system or to reflect minimum levels to avoid depletion. In contrast, in this work, lactate supplementation levels were chosen to specifically eliminate the net production of lactate through the law of mass action. Furthermore, lactate is supplemented into the initial culture medium, rather than as a feed during the lactate consumption phase. The intent is to control cell metabolism from time zero, from inoculation through the full culture duration. If lactate is substantially consumed, it may become necessary to implement lactate supplementation of feeds and/or the use of lactic acid to control pH, such as performed by Li et al. [38].

## 2. Results

### 2.1. Adaptation and Reduced Lactic Acid Production in Shaker Flask Cultures 

**Figure 1:**
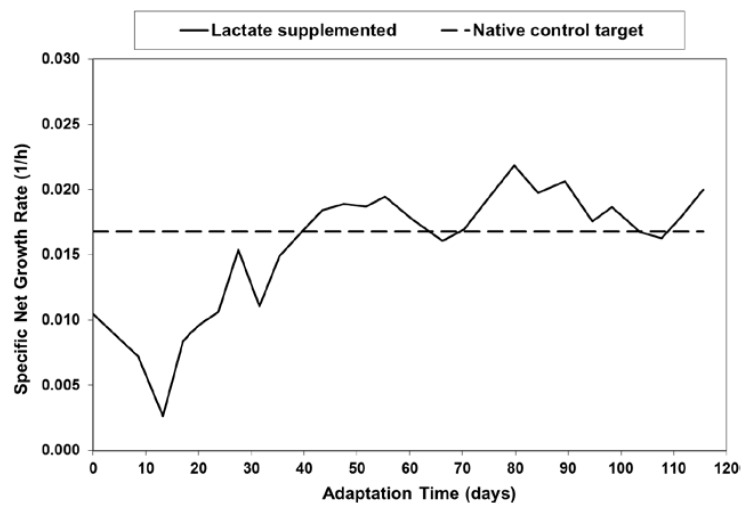
Specific net growth rate in lactate-supplemented medium as a function of adaptation time for batch shaker flask cultures.

As described in the Materials and Methods section, a native control CHO cell line was adapted in shakers to high-lactate in standard Opti-CHO base medium (Invitrogen, Carlsbad, CA, USA) supplemented with sodium l-lactate. The shaker flask cultures were passaged every 3–4 days in lactate-supplemented medium. Figure 1 shows the average specific net growth rate for each passage, along with the native control target, for lactate supplementation levels of roughly 35 mM. As will be presented later, lactate supplementation at 35 mM was sufficient to nearly eliminate the net production of lactic acid. The native control target line in Figure 1 was the average specific net growth rate of the native control cells over many passages.

As shown in Figure 1, when the cells were cultured in the lactate-supplemented medium, they exhibited a reduced specific net growth rate for the first several passages. However, the cells eventually adapted to this culture medium over approximately 40 days, and were subsequently referred to as Lactate-Adapted (LA) cells. Separate native control cultures were adapted to 410 mOsm (via sodium chloride supplementation) and were subsequently referred to as Osmo-Adapted (OA) cells. 

Beyond the adaptation period of 40 days, the LA and OA cells were passaged in shakers in lactate-supplemented or NaCl-supplemented medium, respectively, for an additional 80 days. Both cell types maintained stable growth and phenotype over this time period. The average characteristics are summarized in Table 2. The average specific net growth rates (*μn*) were similar. Statistically significant differences were observed in specific lactic acid production rate (*qLac*), specific glucose uptake rate (*qGluc*), and observed yield of lactate on glucose (*Y_l/g_*), with p values less than 0.001. 

**Figure 2:**
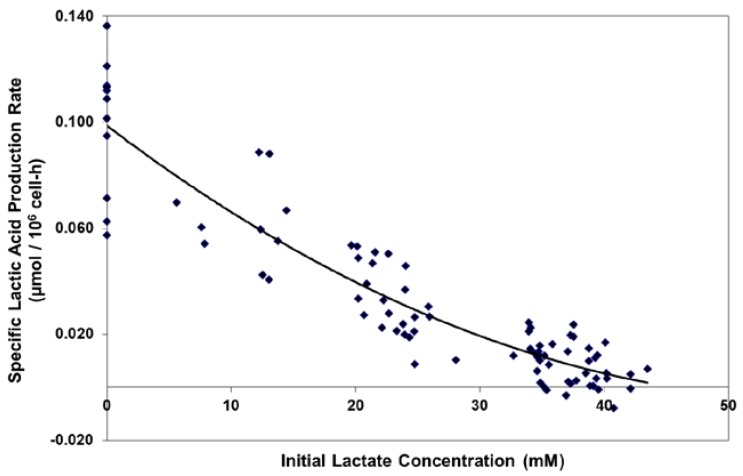
Effect of initial lactate concentration on specific lactic acid production rate in batch shaker flask cultures.

For the Lactate-Adapted cells, as well as the native control cells, increasing levels of l-lactate substantially reduced the specific productivity of lactate, driving it to zero at approximately 40 mM l-lactate (Figure 2). This occurred even though the cells were maintained in exponential growth in shakers at relatively high glucose levels (typically > 10 mM). 

### 2.2. Growth Performance of Adapted Cells in Fed-Batch Bioreactor Cultures 

**Figure 3:**
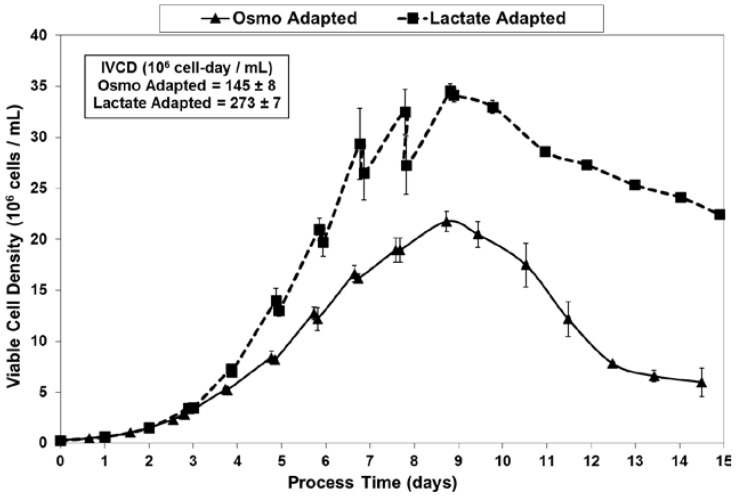
Viable cell concentrations for fed-batch bioreactor cultures.

**Figure 4:**
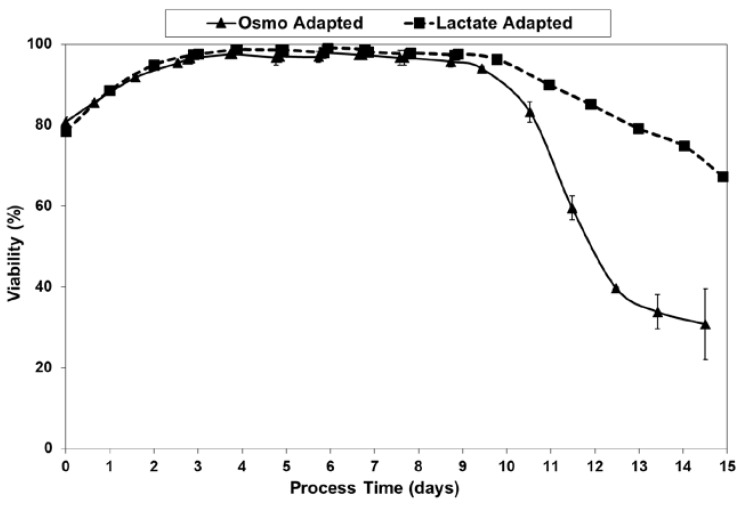
Viabilities for fed-batch bioreactor cultures.

Fed-batch cultures were grown in duplicate 3-L bioreactors using LSA technology (with LA cells) along with control cultures using OA cells. The growth and viability curves for the extended 15 day cultivations are shown in Figure 3 and Figure 4. LA cultures began to outperform OA cultures after the first feed and achieved significantly improved viable cell densities, increasing over one-hundred-fold to reach a maximum cell density of 35 million cells per mL. In contrast, the OA cultures reached a maximum cell density of 22 million cells per mL. The LA cells exhibited substantially higher viabilities during the later stages of culture (Figure 4), followed by a slow death phase. In contrast, the OA cells entered a more rapid death phase after the last feed, ending with a low harvest viability of 30 percent, 40 percentage points below the LA cells. The combination of increased maximum cell density and slow death phase led to a near doubling in Integrated Viable Cell Days (IVCD) for the LA cells as compared to OA cells (Figure 3). The maximum cell density of 35 million cells per mL, as well as the IVCD of 273 million cell-days per mL, as achieved with the LSA technology, are both among the highest values reported in the literature, as will be covered further in the Discussion section.

### 2.3. Lactic Acid Production, Base Addition, and Osmolality Profiles in Fed-Batch Bioreactor Cultures 

**Figure 5:**
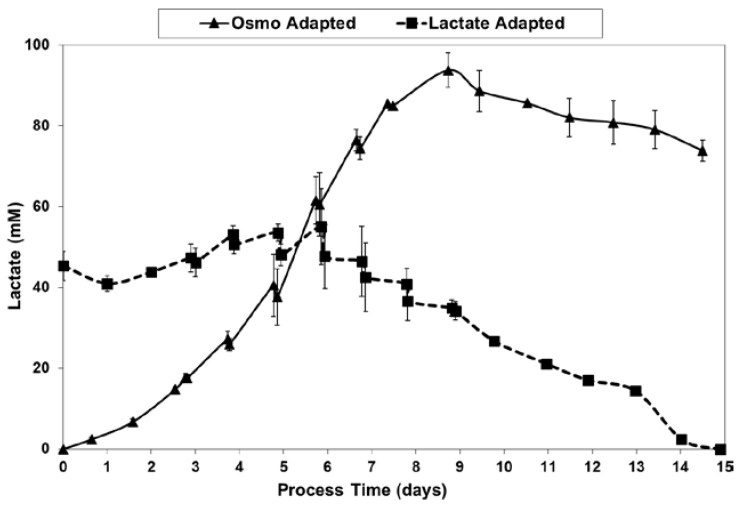
l-lactate levels for fed-batch bioreactor cultures.

**Figure 6:**
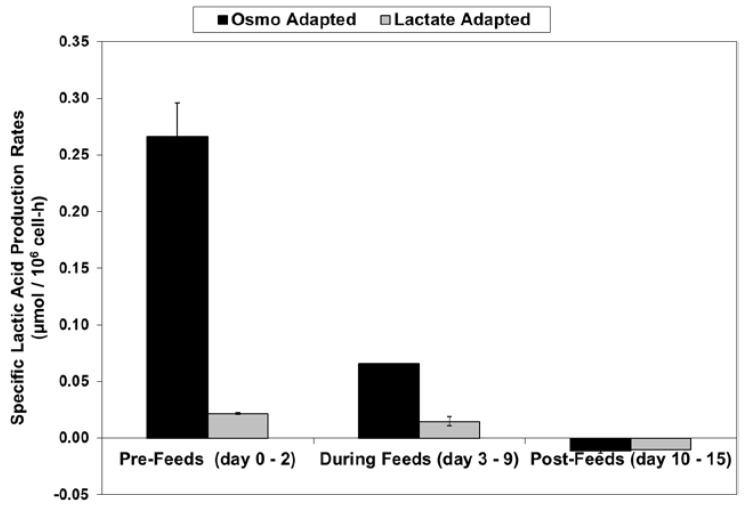
Specific lactic acid production rates for fed-batch bioreactor cultures.

For the fed-batch cultures shown in Figure 3 and Figure 4, Figure 5 shows the total l-lactate concentration, including the initial lactate supplementation level for the LA cells. LSA technology largely eliminated the increase in lactate levels (peak minus initial), from 94 mM for the OA cells down to only 10 mM for the LA cells (9.4-fold decrease). It substantially decreased specific lactic acid production rates from day 0 to day 9 (Figure 6).

**Figure 7:**
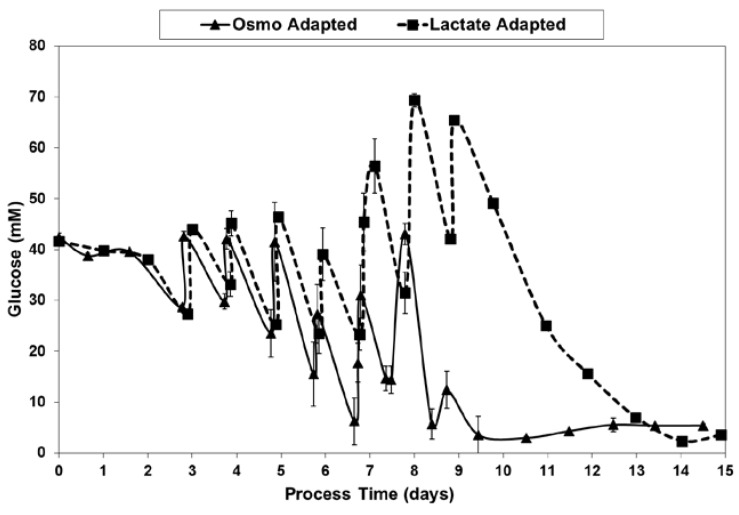
Glucose levels for fed-batch bioreactor cultures.

**Figure 8:**
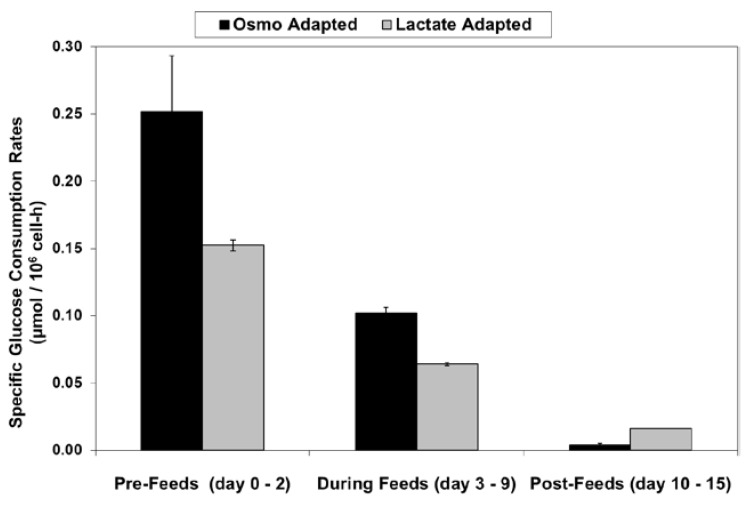
Specific glucose consumption rates for fed-batch bioreactor cultures.

With the LA cells but not the OA cells, very low specific lactic acid production rates were achieved during the entire growth phase, as occurred throughout the “Pre-Feed” and part of the “During Feed” stages shown in Figure 6, even though glucose levels were 20 mM or higher (Figure 7). During the “Post-Feed stage”, the lactate-consumption (LC) phenotype was observed for all cultures. LSA technology provided a partial reduction in specific glucose uptake rates (Figure 8). 

**Figure 9:**
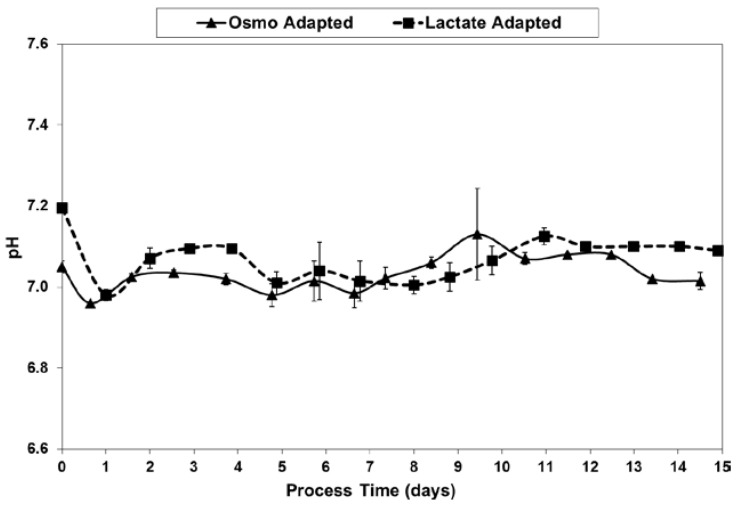
pH levels for fed-batch bioreactor cultures.

As shown in Figure 9, pH levels were well maintained to within 0.1 units of the 7.05 target, after inoculation at 7.05–7.2 and the initial drift down to the dead-band control point. The data is from off-line readings using the Nova BioProfile.

**Figure 10:**
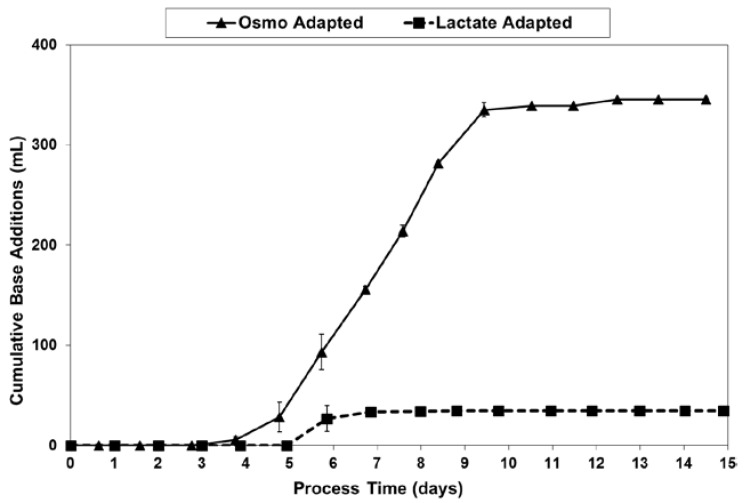
Base addition volumes for fed-batch bioreactor cultures.

LSA technology dramatically reduced the amount of base needed for pH control, from 345–350 mL for the OA cultures down to only 40–45 mL for the LA cultures (Figure 10). This eight-fold reduction in base usage approximately matched the 9.4-fold reduction in lactate increase (peak vs. starting) previously discussed with regard to Figure 5.

**Figure 11:**
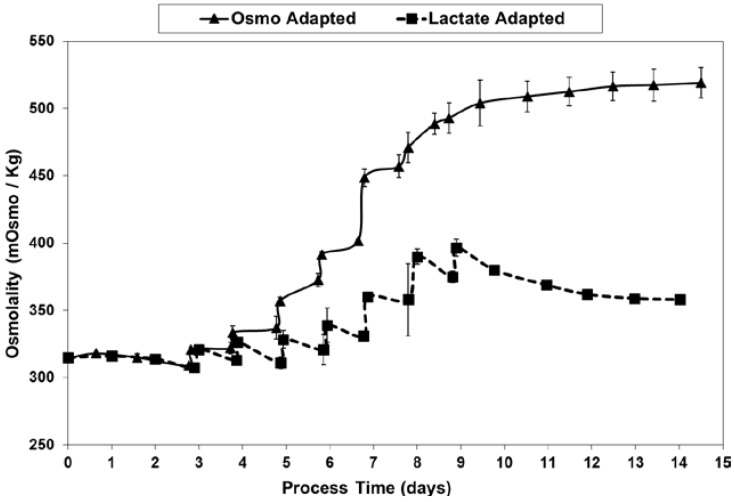
Osmolality levels for fed-batch bioreactor cultures.

At least partly through the dramatic reduction in lactic acid production and associated base additions, LSA technology avoided the excessive increase in osmolality seen for the OA control cultures (Figure 11), likely accounting for at least part of the differences seen in the cell concentration and viability profiles. 

### 2.4. Ammonium Production and Glutamine Consumption in Fed-Batch Bioreactor Cultures 

**Figure 12:**
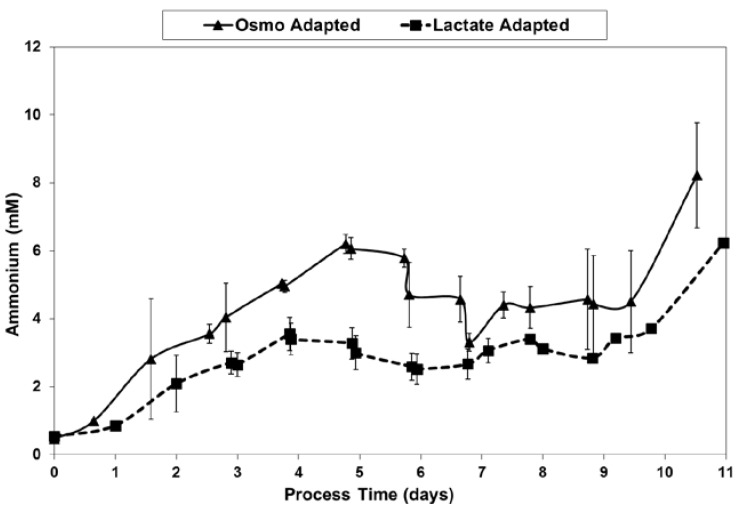
Ammonium levels for fed-batch bioreactor cultures.

**Figure 13:**
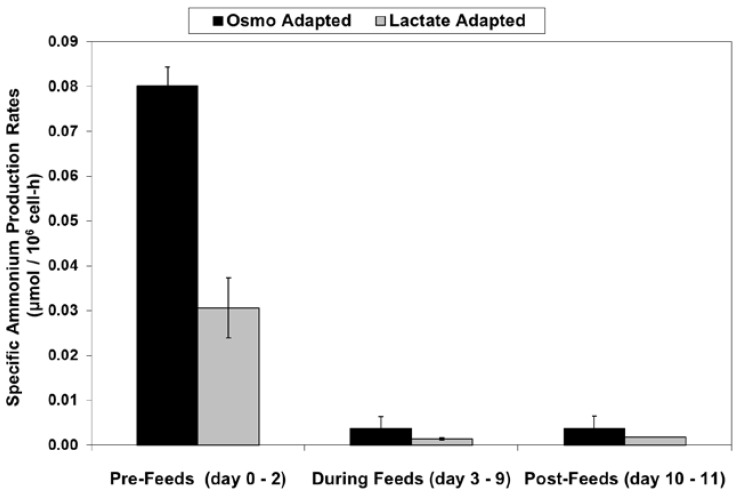
Specific ammonium production rates for fed-batch bioreactor cultures.

LSA technology also resulted in a substantial reduction in ammonium levels (Figure 12), even though cell densities were higher, due to a ~50% reduction in specific ammonium production rates (Figure 13). Average specific glutamine uptake rates were equivalent between the two cell lines. Due to experimental issues, reliable glutamine and ammonium data was available only to day 11, the limit of the data shown in Figure 12 and Figure 13. Error bars shown on Figure 3, Figure 4, Figure 5, Figure 6, Figure 7, Figure 8, Figure 9, Figure 10, Figure 11, Figure 12 and Figure 13 represent the standard deviation between duplicates.

## 3. Discussion

This paper covers the initial feasibility and proof-of-concept studies for a proposed new method to reduce both lactic acid and ammonium production in industrial cell culture. It is a simple method, involving supplementation of culture medium with lactate and adaptation of cells to this medium. For ease of reference, the method has been termed Lactate Supplementation and Adaptation (LSA) technology. 

Using this technology, fed-batch cultures were grown to maximum viable cell density of 35 million cells per mL. This compares well to published values by industry leaders of 5–45 million cells per mL [10,11,27,55,85,86]. Similarly, the achieved IVCD value of 273 million cell-days per mL also compares well to published values by industry leaders of 30–275 million cell-days per mL [11,85,86].

Although LSA technology certainly looks promising, it has only begun to be tested, scrutinized, and optimized. Results were presented in this paper for only one cell line. The technology needs to be tested with more cell lines, including ones that have been genetically engineered to make a monoclonal antibody or other recombinant protein. The impact on specific productivity and product quality needs to be determined for a well-engineered cell line. If the results look promising, it should be tested at larger scales. Combinations of the technology with other promising techniques, such as use of lactate in feed or the use of lactic acid for pH control [38], need to be tested.

Furthermore, proposed mechanisms of action, involving the laws of mass action, were provided to show the rationale behind this new method. Experiments were not conducted to specifically test the proposed mechanisms of action. Such experiments need to be conducted and may lead to further improvement of the method. There may be inhibition of phosphofrutokinase by lactate, as found by Mulukutla et al. [82] for their cultures.

For over 25 years, fed-batch culture of animal cells has been used to manufacture monoclonal antibodies and other recombinant proteins for clinical trial and commercial sales. Over that time period, improvements in the technology and resulting titers have been dramatic [10,87,88,89,90]. Nonetheless, problems with lactic acid and ammonium production persist, in terms of both sporadic problems with current processes [11] and limitations on future progress. Further improvements in fed-batch technology may allow production titers to reach in excess of 20 g/L, thereby permitting economical commercial production of many biopharmaceuticals in single use bioreactors with volumes of only 1000 to 2000 L.

## 4. Materials and Methods 

### 4.1. Native Control Cells, Flask Cultures, and General Culture Methods 

Experiments were conducted with a dihydrofolate reductase deficient (DHFR^−^) Chinese Hamster Ovary cell line, DG44 CHO, ordered from Invitrogen (Invitrogen, Carlsbad, CA, USA) Prior to experimentation, the cells were transfected with a pOPTI-Vec TOPO plasmid vector containing the gene for a recombinant monoclonal ScFv-Fc1 protein fragment. Transfected cells were grown in selective OptiCHO medium (Invitrogen, Carlsbad, CA, USA) and the recombinant gene copy number was further amplified by supplementing the medium with 250 nM methotrexate. The amount of recombinant antibody fragment expressed was less than 10 mg/L and not subsequently measured. This cell line was used to test the feasibility of the proposed method in terms of cell growth, death, and metabolism, but not recombinant protein productivity or product quality. Cells were frozen down and hereafter referred to as “Native Control” (NC) cells. All subsequent experiments were performed without methotrexate.

Flask cultures were grown at 30 mL working volume in 125-mL shaker flasks (Corning, Lowell, MA, USA) placed in an incubator at 37 °C, 135 rpm, and 8% CO_2_. Continuous passages were performed every 3 to 4 days in duplicate shaker flasks inoculated at a seeding density of 1.0 × 10^6^ cell/mL. Cell concentration and viability were measured by the trypan blue exclusion method using a ViCell analyzer (Beckman Coulter, Fullerton, CA, USA). Off-line measurements were taken with a Bioprofile 400 (Nova Biomedical, Waltham, MA, USA) for glucose, glutamine, lactate, ammonium concentration and pH levels. Osmolality was measured by freezing point depression on a Model 3250 Osmometer (Advanced Instruments, Norwood, MA, USA).

### 4.2. Adaptation of Cells

Basal medium was commercially available, serum-free, chemically-defined OptiCHO medium (Invitrogen, Carlsbad, CA, USA), purchased as sterile liquid ready for use, supplemented with 8 mM glutamine (Invitrogen). For use in adaptation, this standard basal medium was supplemented with sodium lactate or sodium chloride, in powder form, prior to sterile filtration. Adaptation to high lactate levels was achieved by continuously passaging the cells in basal medium supplemented with 35 mM sodium lactate (Sigma, Lowell, MA, USA). The starting lactate levels in the culture post seeding as measured by the Bioprofile 400 were 35 ± 10 mM. The measured variability of 10 mM was due to the precision of the Bioprofile measurements as well as differences in lactate levels carried over from seed cultures. Ninety sequential passages over 9 months were performed to monitor stability and run parallel experiments. The cells grown in medium supplemented with roughly 35 mM lactate over a period of at least 40 days, with specific net growth rates during continuous passaging that matched or exceeded the rates for Native Control cells in medium without lactate, are referred to as “Lactate Adapted” (LA) cells. Osmolality levels were 400–410 mOsmo. Lactate levels specified throughout this paper, including those measured by the Nova Bioprofile 400, are only for the l-isomer. 

35 mM lactate was ultimately chosen as the target supplementation during adaptation sufficient to drive lactic acid production to near zero. A range of 0 to 40 mM was investigated, based on the typical peak amounts produced in fed-batch culture for the cell line used and observation of the lactate-consumption phenotype in the later stages of such cultures. Supplementation above 40 mM was not studied as the desired effect was achieved within the range tested. 

In a similar fashion, a cell line adapted to high osmolality was derived through continuous passaging in medium supplemented with sodium chloride (Sigma, Lowell, MA, USA). The level of sodium chloride was chosen to provide a matching osmolality level of 400–410 mOsmo. The cells grown in medium supplemented with this sodium chloride level over a period of at least 40 days, with specific net growth rates during continuous passaging that matched or exceeded the rates for Native Control cells, are referred to as “Osmo Adapted” cells. 

### 4.3. Bioreactor Cultures

Bioreactor cultures were grown in 3 L Applikon bioreactors (Foster City, CA, USA) with an initial working volume of 1.6 L. All conditions were tested in duplicate. Each bioreactor consisted of a round-bottom glass vessel and stainless steel head plate configured with sparging, overlay, and exhaust ports, five medium addition ports, a sampling port, base and antifoam inlet ports, and pH, dissolved oxygen (DO) and temperature probe ports. A 3-bladed, pitched-blade impeller (called a marine impeller by Applikon) was set at 250 rpm between days 0 and 3 then increased to 300 rpm on day 4. Temperature was controlled at 37 °C through the use of a heating blanket, while pH was controlled at 7.05 with a dead band of 0.03 using sparged CO_2_ gas or 1.0 N (0.5 M) sodium carbonate (Ricca Chemical Company, Arlington, TX, USA). Dissolved oxygen (DO) was maintained at 50% air saturation through the use of the standard Applikon drilled tube sparger. The sparge gas was air between days 0–4 and then switched to oxygen on day 5. Solenoid valves were used for on/off control of the sparge gas. Air sparge gas flow rate was controlled at 500 mL/min while oxygen sparge was controlled at 100 mL/min, both with rotameters. An air overlay was also used. 

As determined through readings from the Nova Bioprofile for days 0–7, prior to sporadic issues with such measurements from day 8 onward, pCO_2_ levels were comparable between the LA and OA bioreactors, dropping from roughly 90 mm Hg on day 1 down to roughly 10 mm Hg on days 4–5, then back up to roughly 50 mm Hg on day 7. From roughly day 11 onward, pCO_2_ levels were elevated up to 90–140 mm Hg due to the use of CO_2_ sparging for pH control. There was very little to no base addition to either set of cultures from day 9 onward. 

To initiate cultures, bioreactors were first batched with basal medium, allowed to reach temperature and DO set points, and then inoculated at a seeding density of 0.3 × 10^6^ cells/mL. Antifoam B (Sigma, Lowell, MA, USA) was added as needed to eliminate foaming, in increments of 1 mL/L at 10% concentration. 

### 4.4. Fed-Batch Experiments

To reduce starting osmolality and thus allow for more additions of concentrated fed-batch feeds, a custom version of the OptiCHO basal medium was used. This custom medium was ordered free of glucose and NaCl and then supplemented with 8 mM glutamine and 5.8 g/L glucose (Invitrogen, Carlsbad, CA, USA). It was further supplemented with 40 mM Na-lactate for the LA cell line or 40 mM NaCl (Sigma, Lowell, MA, USA) for the OA cell line. For both cell lines, the resulting osmolality of the basal medium was 315 mOsmo. 

Table 3 details the concentrated nutrient feed solutions used for the fed-batch experiment. One set of nutrient feeds was prepared by adding CD CHO AGT complete medium powder (Invitrogen) to 1× Efficient Feed B liquid formulation (Invitrogen). For feed solutions HCD-1 and HCD-2, CD CHO AGT powder was added at levels of 12 and 24 g/L, respectively, to Efficient Feed B. An additional 40 mL/L of 200 mM glutamine was also added to each feed solution. The resulting glucose levels were 21 g/L and 24 g/L, respectively, for HCD-1 and 2. Two additional feed solutions, HCD-3 and HCD-4, were prepared, as shown in Table 3. Sodium lactate was not added to the feeds. 

Table 4 describes the feeding schedule of the solutions listed in Table 3. The schedule was based on the assumption that the proprietary Efficient Feed B and CD CHO AGT formulations from Invitrogen were stoichiometrically-balanced, wherein the nutrient levels are present in proportion to their relative uptake rates. Accordingly, feed volumes were selected upon the basis of maintaining a sufficient concentration of glucose, a key nutrient readily measured with the Nova Bioprofile device. Every 24 h the cultures were sampled and glucose levels measured. A sufficient volume of concentrated nutrient feeds was added so that the glucose levels would ideally remain above 10 mM over the next 24 h period. In practice, glucose levels occasionally dropped slightly below 10 mM, but were never below 5 mM. On any given day, the same feeds and feed volumes were added to all four bioreactors, according to the schedule shown in Table 4. Glucose uptake in the OA cultures was often higher than in the LA cultures, and thus the glucose levels in the OA cultures were used to determine the feed volumes. Feeds were continued until the maximum practical working volume of nearly 3 L was reached on day 9 for the OA cultures. By that time, over 11% of the volume (345–350 mL) in the OA cultures was due to base addition. Although there was still ~300 mL of space available in each LA culture, no additional feeds were added. 

### 4.5. Calculation of Specific Rates

Specific rates were determined on a time-interval basis by measuring daily viable and total cell density (*VCD* or *TCD*) and metabolite concentrations. Specific net growth rate (*μ_N_*), was calculated as change in *VCD* over a time interval *t*_1_ to *t*_2_ using Equation (5):

(5) μN=ln[VCD2VCD1]t2−t1

Specific total growth rate (*μ_T_*) and death rate (*Kd*) were determined in the same time interval using Equations (6)–(8):

(6) $$\mu_{T}=\mu_{N}(\frac{TCD_{2}-TCD_{1}}{VCD_{2}-VCD_{1}})\;\mathrm{for}\;VCD_{1}\neq VCD_{2}$$

(7) μT=(TCD2−TCD1VCD1t2−t1) for VCD1=VCD2

(8) $$Kd=\mu_{T}-\mu_{N}$$

Metabolic flux was calculated as specific nutrient consumption rate or specific metabolite production rate (*q_p_*) using Equations (9) and (10), where *P* is nutrient (glucose or glutamine) or metabolite (lactic acid or ammonium) concentration:

(9) $$q_{P}=\mu_{N}\left(\frac{P_{2}-P_{1}}{VCD_{2}-VCD_{1}}\right)\;\mathrm{for}\;VCD_{1}\neq VCD_{2}$$

(10) qP=(P2−P1VCD1t2−t1) for VCD1=VCD2

Integral Viable Cell Days [87] was calculated via the trapezoid method. Error bars shown on graphs indicate one standard deviation each direction. JMP^®^ software was used for statistical analysis. 

## 5. Patents

The two authors are co-inventors on a patent covering LSA technology (U.S. 8,470,552 B2). Interested parties are encouraged to contact Professor Croughan regarding (a) its availability for license, and (b) results from studies conducted beyond the work shown in this publication.

## Figures and Tables

**Table 1:** Common methods to reduce lactic acid and/or ammonium production in animal cell culture.

Method	References	Effect on Lactic Acid (Lac A) Production	Effect on Ammonium (NH_4_^+^) Production	Likely Universally Reduces Lac A and NH_4_^+^ Production?	No Change or Decrease in Process Complexity?	Commonly ImplemenTed in cGMP Operations?
Replacement of glucose (gluc), glutamine (gln), or both with alternative sugars and/or amino acids	[1,2,13,14,15,16] (gluc only)	Reduced	NR	No	Yes	No
	[17,18] (gln only)	NR	Reduced	No	Yes	No
	[3,19,20] (gluc only)	Reduced	Increased	No	Yes	No
	[21] (gluc only)	Reduced	Unchanged	No	Yes	No
	[20] (both)	Reduced	Reduced	Possibly	Yes	No
On-line feedback control of glucose and/or glutamine at very low levels using glucose sensor, glutamine sensor, and/or other sensors and concentrated feeds	[22,23,24] (gluc only)	Reduced	Increased	No	No	No
	[23] (gln only)	Reduced	Reduced	No	No	No
	[4,22,23,24,25,26] (both)	Reduced	Reduced	Yes	No	No
	[3,27] (gluc only with other sensors)	Reduced	Increased	No	No	27 at Pfizer
	[28] (both with other sensors)	Reduced	Unchanged	Yes	No	Not in completely full form
Copper supplementation	[12,29,30,31,32]	Reduced	NR	No	Yes	Yes
Reduction in temperature	[33]	Reduced	Unchanged	No	Yes	Yes
	[34]	Reduced	Reduced	No	Yes	Yes
Reduction in pH	[34,35,36]	Reduced	Unchanged	No	Yes	Yes
	[37]	Reduced	Increased	No	Yes	Yes
Selection of clones with lactate consumption phenotype	[10,11,12,27,30,38]	Reduced	Mixed	No	Yes	Yes

NR—not reported

**Table 2:** Average net growth and metabolic rates of adapted cells in batch shaker flask cultures.

Cell—Line	*μ_n_* (*p* ≈ 0.01) (1/h)	*q_Lac_* (*p* < 0.001) (μmol/10^6^ Cell-h)	*q_Gluc_* (*p* < 0.001) (μmol/10^6^ Cell-h)	*Y_l/g_* (*p* < 0.001)
Lactate Adapted	0.025 ± 0.001	0.022 ± 0.018	0.054 ± 0.006	0.388 ± 0.344
Osmo Adapted	0.027 ± 0.002	0.112 ± 0.032	0.078 ± 0.012	1.411 ± 0.284

*μ_n_*—specific net growth rate, *qLac*—specific lactic acid production rate, *qGluc*—specific glucose consumption rate, *Y_l/g_*—lactate produced per glucose consumed.

**Table 3:** Concentrated nutrient feed solutions.

Feed Solution	Component Levels in Given Feed Solution				Measured (*) or Theoretical (+) Osmolality
	Efficient Feed B	CD CHO AGT	Glutamine	Glucose	
HCD-1	1×	12 g/L	8 mM	21 g/L	540 mOsmo *
HCD-2	1×	24 g/L	8 mM	24 g/L	700 mOsmo *
HCD-3	-	-	200 mM	-	200 mOsmo ^+^
HCD-4	-	-	300 mM	500 g/L	3080 mOsmo ^+^

**Table 4:** Concentrated nutrient feed schedule.

Feed Solution	Day 2	Day 3	Day 4	Day 5	Day 6	Day 7	Day 8	Day 9
HCD-1		80 mL	80 mL	80 mL	160 mL			
HCD-2						320 mL	185 mL	80 mL
HCD-3				16 mL	16 mL	32 mL		
HCD-4							20 mL	20 mL

## Abbreviations

cGMP	current Good Manufacturing Practice(s)
CHO	Chinese Hamster Ovary
gln	glutamine
gluc	glucose
GS	Glutamine Synthetase
GS-CHO	Glutamine Synthetase transfected Chinese Hamster Ovary
HIPDOG	HI-end pH-controlled Delivery of Glucose
IVCD	integrated viable cell days
LA	lactate adapted cells
Lac	lactate
Lac A	lactic acid
Lac_i_	initial lactate
LC	lactate consumption
LSA	Lactate Supplementation and Adaptation
NH_4_^+^	ammonium
OA	osmolality adapted cells
NR	not reported
P	nutrient or metabolite
*q_Gln_*	specific glutamine uptake rate in μmol/10^6^ cells-h
*q_Gluc_*	specific glucose uptake rate in μmol/10^6^ cells-h
*q_Lac_*	specific lactic acid production rate in μmol/10^6^ cells-h
*q_NH_4_^+^_*	specific ammonium production rate in μmol/10^6^ cells-h
TCA	tricarboxylic acid
TCD	Total Cell Density
VCD	Viable Cell Density
*Y_l/g_*	lactate produced per glucose consumed (mole/mole)
*K_d_*	specific death rate in h^−1^
*μ_N_*	specific net growth rate in h^−1^
*μ_T_*	specific total growth rate in h^−**1**^
